# Framework for Leadership and Training of Biosafety Level 4 Laboratory Workers

**DOI:** 10.3201/eid1411.080741

**Published:** 2008-11

**Authors:** James W. Le Duc, Kevin Anderson, Marshall E. Bloom, James E. Estep, Heinz Feldmann, Joan B. Geisbert, Thomas W. Geisbert, Lisa Hensley, Michael Holbrook, Peter B. Jahrling, Thomas G. Ksiazek, George Korch, Jean Patterson, John P. Skvorak, Hana Weingartl

**Affiliations:** University of Texas Medical Branch, Galveston, Texas, USA (J.W. Le Duc, M. Holbrook); National Biodefense Analysis and Countermeasures Center, Frederick, Maryland, USA (K. Anderson, J.E. Estep); National Institutes of Health, Hamilton, Montana, USA (M.E. Bloom); Public Health Agency of Canada, Winnipeg, Manitoba, Canada (H. Feldmann); Boston University School of Medicine, Boston, Massachusetts, USA (J.B. Geisbert, T.W. Geisbert); US Army Medical Research Institute of Infectious Diseases, Fort Detrick, Frederick (L. Hensley, G. Korch, J.P. Skvorak); National Institutes of Health, Bethesda, Maryland, USA (P.B. Jahrling); Centers for Disease Control and Prevention, Atlanta, Georgia, USA (T.G. Ksiazek); Southwest Foundation for Biomedical Research, San Antonio, Texas, USA (J. Patterson); Canadian Food Inspection Agency, Winnipeg (H. Weingartl)

**Keywords:** BSL-4 laboratory, containment laboratories, training, perspective

## Abstract

Construction of several new Biosafety Level 4 (BSL-4) laboratories and expansion of existing operations have created an increased international demand for well-trained staff and facility leaders. Directors of most North American BSL-4 laboratories met and agreed upon a framework for leadership and training of biocontainment research and operations staff. They agreed on essential preparation and training that includes theoretical consideration of biocontainment principles, practical hands-on training, and mentored on-the-job experiences relevant to positional responsibilities as essential preparation before a person’s independent access to a BSL-4 facility. They also agreed that the BSL-4 laboratory director is the key person most responsible for ensuring that staff members are appropriately prepared for BSL-4 operations. Although standardized certification of training does not formally exist, the directors agreed that facility-specific, time-limited documentation to recognize specific skills and experiences of trained persons is needed.

## Abstract 2

One-sentence summary for table of contents: Training should include theoretical consideration of biocontainment principles, practical hands-on training, and mentored on-the-job experience.

The threat of terrorists using as weapons the most deadly pathogens known, coupled with the recognition that virtually every year a new infectious disease is discovered, has led the US government to expand the number of Biosafety Level 4 (BSL-4) laboratories, also known as maximum containment laboratories (MCLs), to perform work essential for promoting public health and to ensure bioterrorism preparedness. A few such laboratories have been in existence for decades, primarily in Australia, Russia, South Africa, the United Kingdom, and the United States; these have had, in most instances, an exceptional history of safety in handling these most dangerous pathogens. However, construction of new facilities, including 2 national laboratories on academic campuses in the United States, and the expansion of existing US facilities, has resulted in public concern and Congressional inquiries regarding the safety of these laboratories and the qualifications of those persons working in them (*1**,**2*).

Historically, a small, close-knit group of dedicated scientists have made their careers working in this highly specialized BSL-4 environment. Laboratory directors have generally had hands-on involvement in research activities undertaken in their facilities, have personally trained those with whom they worked, and have been careful to restrict access to those not well suited for containment-laboratory endeavors. The current expansion of BSL-4 laboratories, within the United States and in several other countries, has resulted in a demand for experienced workers, which presents a unique challenge to currently established processes for BSL-4 training (*3*). Given the global demand for BSL-4 laboratory workers to staff the expanded number of facilities, there is a clear international need for more structured and transparent BSL-4 training processes to establish and verify standards for the next generation of containment-laboratory scientists (*4*). Development of rigorous standards for BSL-4 laboratory training will instill confidence in the public, policy makers, and security officials that the expanded international network of BSL-4 laboratories will continue to be operated safely and will pose no risk to scientific staff, local communities, surrounding environment, and host nations. Clarification and coordination of training standards will help to develop a cadre of highly qualified biocontainment workers and will result in a series of robust BSL-4 laboratory programs that will enable scientists to develop measures to deal with existing threat agents and to cope with new diseases that emerge (*5*).

In response to this challenge, the BSL-4 laboratory directors from most existing North American laboratories and those currently under construction met to develop a framework of standards and norms necessary for training future MCL scientists and support staff. The results of those deliberations are summarized below and offered as a model for the global BSL-4 laboratory community.

## MCL Management Structure

Although the institutional director (e.g., director of the Centers for Disease Control and Prevention, commander of the US Army Medical Research Institute of Infectious Diseases, or dean at an academic campus) has ultimate responsibility for the BSL-4 facility housed within his or her institution, it is the BSL-4 laboratory director that senior leadership relies upon to ensure that this unique facility operates safely and securely. The BSL-4 program director oversees all personnel working in the containment laboratory, ensures proper training and qualifications for the work to be undertaken, ensures that all regulatory requirements are addressed, and is responsible for maintaining a safe and efficient work environment. The BSL-4 laboratory director often also serves as the lead scientist for investigations involving highly pathogenic organisms, sets priorities and coordinates activities within the facility, and frequently serves as the technical spokesperson for the program. The BSL-4 laboratory director grants final approval for personnel to operate independently in the BSL-4 laboratory. His or her efforts are complemented by those of the institutional biosafety officer or manager, who plays an important role as an independent advisor to the institutional director to ensure the safe and secure operations of the program, and the building engineer, who manages the complex mechanical infrastructure necessary to enable safe handling of highly dangerous pathogens. The BSL-4 laboratory director works in close partnership with these professionals to ensure smooth operation of the MCL. Each person has distinct responsibilities and, in most instances, a parallel reporting chain that ensures that problems are brought to the level of the institutional director or the academic dean for resolution.

### Preparation of a Person for BSL-4 Work

Persons seeking access to a BSL-4 laboratory come from many different backgrounds, but they must all share common traits of having an aptitude for work with infectious agents and an appreciation of the need for careful adherence to safety standards and protocols. Medical examinations, security checks, and clearances are required before a person can handle select agents; some laboratories require vaccinations before a person can begin work with an infectious agent (licensed vaccines are not widely available for most BSL-4 pathogens.) There is a need to remain flexible in the selection of persons for BSL-4 training, recognizing that some persons rapidly acquire the skills needed to work safely in the BSL-4 laboratory, while others may never gain complete confidence of the MCL director and will always be required to work in partnership with a more experienced person. Prior work at a BSL-3 laboratory is generally considered a strong asset but is not an absolute requirement before being cleared to work in a BSL-4 laboratory. What is essential is that the person must be properly trained in the techniques he or she will be using in the BSL-4 laboratory.

In addition to core scientific staff, there is an ongoing need for BSL-4 laboratory support personnel to service equipment, maintain the building, conduct inspections, and assist in specific technical activities such as the care and use of laboratory animals. These persons also require specialized training and approval to operate independently.

## Elements of Training

Formal training in preparation for work in a BSL-4 laboratory should consist of 3 elements: didactic or classroom-style theoretical preparation, one-on-one practical training in the facility, and mentored on-the-job training (Figure). Theoretical training helps laboratory workers develop an understanding of the underpinnings of biocontainment operations and the laboratory systems that support these operations. Hands-on practical training includes a comprehensive orientation to the specific facility in which the person will work to include a complete review and documented understanding of all standard operating procedures; orientation to engineering aspects of the facility; overview of all safety procedures, including alarms and emergency operations; and an introduction to the care and use of a protective suit or glove box. The institutional biosafety officer and building engineer typically assist in providing this orientation, some of which may be augmented by training videos.

**Figure:**
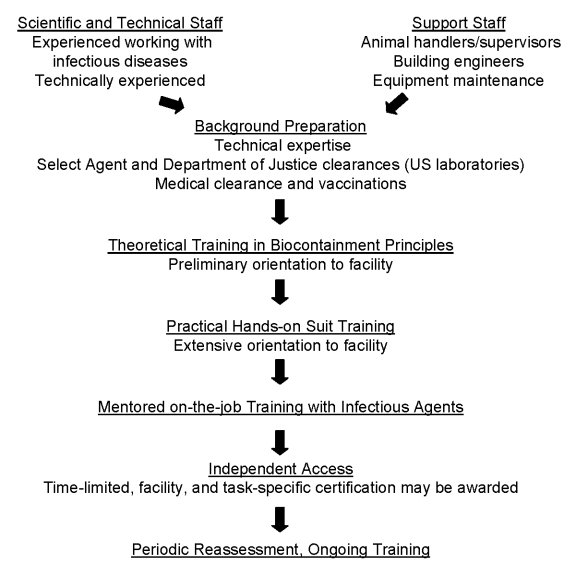
Framework for maximum containment laboratory training.

BSL-4 laboratory orientation training assumes that the person has already mastered all procedures for safe and secure handling of infectious agents at the BSL-2 and ideally BSL-3 levels. This training generally involves individualized orientation within the facility provided by an experienced staff member or dedicated training officer. It may begin while the laboratory is shut down for annual recertification and maintenance or while it is operational. Training would involve use of entrances simply designed to demonstrate how one enters and exits the suite, general orientation on the use of air hoses, working within biologic safety cabinets or glove boxes, storage and record keeping of pathogens, clean-up and decontamination following procedures or spills, solid and liquid waste management, use of autoclaves and other specialized equipment, communications with others inside and outside of the BSL-4 facility, and other general procedures.

Finally, the person under consideration is assigned a dedicated mentor and is introduced to working with live pathogens in the BSL-4 laboratory under the mentor’s close supervision. This stage of training is basically open ended; the length of time and number of entries into the facility will vary greatly depending upon the skills of the person and his or her ability to master all procedures necessary for independent work. Final decision of when a person is allowed independent access is subjective and based on an assessment by the mentor and laboratory director; it is usually discussed only after the person has had extensive experience working in the facility. The time required to gain full independent access may also vary depending upon the kind of work that person will be undertaking. For example, persons not likely to be directly handling infectious material, such as safety officers, building engineers, or maintenance staff, may be offered limited independent access sooner than a person who will be handling live pathogens routinely. Partial or limited access may also be granted to a person for independent access only during normal duty hours. Laboratory procedures that involve animals or sharp instruments (e.g., needles, syringes, postmortem procedures) represent the greatest risk and consequently require special training and experience; these procedures should be mastered at lower containment levels before a person is permitted to undertake these activities under BSL-4 conditions. Most standard operating procedures for animal manipulation require that at least 2 persons be present, regardless of their experience level. Some laboratories require a final oral or written examination before granting a person independent access, which may be administered by the safety officer. However, the ultimate decision as to who is allowed independent access to the BSL-4 laboratory is made by the BSL-4 laboratory director.

A typical mentor will be an experienced person who has earned full unrestricted access to the laboratory and has the clear confidence of the laboratory director. Although there are no set time or formal educational requirements to become a mentor, mentors should have extensive practical experience working under BSL-4 laboratory conditions.

All laboratories should have developed a process for reevaluation of all persons working in the BSL-4 laboratory to ensure that their knowledge and skills remain current. This process may be an annual refresher course or periodic formal or informal review and training and may be augmented by orientation sessions as new equipment is introduced into the facility. Ensuring that senior program staff members are regularly present in the laboratory is important for maintaining consistent safety, security, and scientific standards.

## Need for Certification of Training

The need to document that a person has completed appropriate training has been discussed extensively. It is evident that a tacit internal certification exists in BSL-4 facilities currently operating and this takes the form of approval to work independently. This certification may be more formally captured in a specific document or may be a checklist signed by the approval authority. A more broadly applicable documentation system could provide evidence of consistency in training, demonstrate recognized capabilities with certain tasks such as for animal handlers, and provide a mechanism to gauge the number of persons working in the field.

At present, those working in BSL-4 laboratories in the United States need security clearance and approval to handle select agents, must have completed the extensive training program described above, must have medical examinations, and must be known by the program director. Each BSL-4 laboratory is, however, unique and every program director should demand that all persons entering their facility be well prepared and knowledgeable of all safety and security procedures required of that facility. Although standardized documentation of training does not formally exist, there would be merit in developing an internationally agreed-upon facility-specific, time-limited document to recognize the specific skills and experiences of a person. Such documentation would have the added benefit of facilitating collaborations and personnel exchanges among BSL-4 laboratories.

## Conclusions

Directors of most North American existing and proposed BSL-4 laboratories agreed upon a framework for training of BSL-4 laboratory staff, including scientific, technical, and support personnel such as animal handlers, building engineers, and maintenance workers. Independent access to the BSL-4 facility would be granted at the discretion of the BSL-4 program director after successful completion of training in the theory of biocontainment principles, practical hands-on suit training, and extensive supervised work with infectious agents under the tutelage of a well-experienced mentor. Periodic reassessment of skills and ongoing refresher training would be a routine aspect of continuing education for all BSL-4 staff. The need for documentation of training that would be time-limited and specific for a given facility was also discussed. Such formal documentation could facilitate collaborations and personnel exchange between BSL-4 facilities and help to better certify the national BSL-4 workforce. The framework proposed could serve as a model for BSL-4 workforce development globally.
